# Retraction Note: Acute Toxicity and Gastroprotection Studies of a New Schiff Base Derived Manganese (II) Complex against HCl/Ethanol-Induced Gastric Ulcerations in Rats

**DOI:** 10.1038/s41598-020-63217-y

**Published:** 2020-04-17

**Authors:** Mohamed Yousif Ibrahim, Najihah Mohd Hashim, Summaya M. Dhiyaaldeen, Mazen M. Jamil Al-Obaidi, Rashd M. El-Ferjani, Hoyam Adam, Bassam Alkotaini, Rami Al Batran, Hapipah Mohd Ali

**Affiliations:** 1 Department of Pharmacy, Faculty of Medicine, University of Malaya, 50603 Kuala Lumpur, Malaysia; 2 Department of Chemistry, Faculty of Science, University of Malaya, 50603 Kuala Lumpur, Malaysia; 3 Center for Natural Products and Drug Discovery (CENAR), University of Malaya, Kuala Lumpur, Malaysia; 4 Department of Microbiology, Faculty of medicine, University of Duhok, 78 Kurdistan, Iraq; 5 Department of Biomedical Science, Faculty of Medicine, University of Malaya, 50603 Kuala Lumpur, Malaysia; 6 Department of Chemistry, Faculty of Science, University of Benghazi, 1308 Benghazi, Libya; 7 School of Pharmacy, Ahfad University for Women (AUW), 167 Omdurman, Sudan; 8 Department of Chemical Engineering, Faculty of Engineering, Chungbuk National University, Cheongju, Chungbuk 28644 Republic of Korea; 9 Institute of Research Management & Monitoring, Deputy Vice Chancellor (Research & Innovation), University of Malaya, 50603 Kuala Lumpur, Malaysia

Retraction of: *Scientific Reports* 10.1038/srep26819, published online 27 May 2016

The Editors have retracted this Article.

An investigation at the University of Malaya identified figure assembly and data integrity issues. Specifically, the following images were duplicated from a previous publication [1]: Fig. 6 G3 (Hsp70 stain, omeprazole (20mg/kg)) and Fig. 6 G4 (Hsp70 stain, MDLA (500mg/kg)) were duplicates of Fig. 9E (HSP70 stain, omeprazole (20mg/kg)) and Fig. 9C (HSP70 stain, HPTP (50mg/kg)), respectively. These issues undermine confidence in the study which cannot be considered reliable.

Najihah Mohd Hashim, Mazen Al-Obaidi, Bassam Alkotaini, and Rami Al Batran agree with the retraction. The other authors did not respond to correspondence about this retraction.

## References

[1] Dhiyaaldeen SM (2014). Protective effects of (1-(4-hydroxy-phenyl)-3-m-tolyl-propenone chalcone in indomethacin-induced gastric erosive damage in rats. BMC Vet Res.

